# Striking a Balance: Work-Health-Personal Life Conflict in Women and Men with Arthritis and its Association with Work Outcomes

**DOI:** 10.1007/s10926-013-9490-5

**Published:** 2013-12-27

**Authors:** Monique A. M. Gignac, Diane Lacaille, Dorcas E. Beaton, Catherine L. Backman, Xingshan Cao, Elizabeth M. Badley

**Affiliations:** 1 Institute for Work and Health, 481 University Avenue, Suite 800, Toronto, ON M5G 2E9 Canada; 2 Division of Health Care and Outcomes Research, Arthritis Community Research and Evaluation Unit, Toronto Western Research Institute, Toronto, ON Canada; 3 Dalla Lana School of Public Health, University of Toronto, Toronto, ON Canada; 4 Department of Medicine, Arthritis Research Centre of Canada, University of British Columbia, Vancouver, BC Canada; 5 St. Michael’s Hospital, Toronto, ON Canada; 6 Health Policy, Management and Evaluation, University of Toronto, Toronto, ON Canada; 7 Occupational Science and Occupational Therapy, University of British Columbia, Vancouver, BC Canada; 8 Arthritis Research Centre of Canada, Vancouver, BC Canada; 9 Arthritis Community Research and Evaluation Unit, Toronto Western Research Institute, Toronto, ON Canada

**Keywords:** Employment, Arthritis, Role concept, Role balance, Work-family conflict, Role overload

## Abstract

*Purpose* To examine men and women’s perceptions of inter-role balance/imbalance in work, arthritis, and personal roles and its association with demographic, health and employment factors, including job stress, career satisfaction, job disruptions, absenteeism and perceived productivity losses. *Methods* Participants were employed, aged ≥40 years and diagnosed with osteoarthritis or inflammatory arthritis. They were recruited through community advertising and rheumatology clinics in two Canadian provinces. Respondents completed a 35–45 min telephone interview and a 20-min self-administered questionnaire assessing role perceptions [(arthritis negatively impacts work (A → W); work/personal life negatively impact arthritis (W/P → A); work as a positive role (W +))], demographic, health and work context information. Analyses included exploratory factor analysis and multivariate regressions. *Results* Findings revealed similarities between men (*n* = 104) and women (*n* = 248) in health, work and role perceptions, although women reported more benefits of working with arthritis (W+) than men. Some gender differences were found in factors associated with inter-role perceptions highlighting the importance of children, fatigue, unpredictable work hours, job control, and workplace activity limitations. Role perceptions were associated with work outcomes but only one perception, W/P → A, interacted with gender. Among men, greater perceptions that work and personal demands interfered with managing arthritis were associated with more job disruptions. *Conclusions* This study revealed negative and positive inter-role perceptions related to working with a chronic illness and associations with work outcomes. It highlights potentially modifiable factors that could assess risk and inform interventions to improve role balance and working experiences.

## Introduction

For most workers, employment occurs in conjunction with other role demands related to family and personal relationships, household tasks and social and leisure activities. To date, a considerable number of studies have examined work-personal life balance. Findings indicate that perceptions of inter-role conflict and role overload are common among employed women and men and are associated with work and health outcomes like reduced job satisfaction and lower work performance, as well as increased work absenteeism, job turnover, depression and burnout [1–9]. However, few studies have examined role balance in the context of chronic disease. This oversight is important as chronic conditions often affect individuals in their prime working years when they have multiple role demands [10]. Many chronic conditions also increase in prevalence with age and will gain in importance with the greying of the workforce that is underway in many developed countries [11–15]. A better understanding of the nexus among work, chronic disease, and personal life can help identify individuals at risk for difficulties sustaining employment and inform ways to manage chronic diseases in the workplace.

Among the most prevalent chronic health conditions affecting employment are rheumatic diseases like arthritis [16–20]. Arthritis is associated with giving up work, increased sick leave, absenteeism and at-work productivity loss (presenteeism) [19, 21–30]. Challenges in working with arthritis include dealing with ongoing or intermittent disease symptoms like pain and fatigue, as well as activity limitations with work tasks [31–35]. Work context factors such as physically demanding work, a high work pace, low job control, and commuting also can make working problematic for people with the disease [33, 35–41].

In addition to the potentially negative impact of arthritis on work, a small number of studies have examined whether individuals perceive that their job or their personal life affects their health or management of their disease [35, 38, 42]. For example, a qualitative study of individuals with inflammatory arthritis (e.g., rheumatoid arthritis) and osteoarthritis found that many individuals reported both negative and positive aspects of working with arthritis. Negative comments highlighted that arthritis symptoms or their treatment sometimes interfered with performance of job tasks. But the opposite direction was also true with individuals noting that work and personal demands could interfere with health care appointments or was exhausting, making it difficult to find time and energy to optimize care of their disease [42]. Despite these difficulties, individuals also reported positive aspects of working with arthritis. Maintaining employment was highly valued. This was not only because of financial resources and access to benefits that jobs provided (e.g., medication, extended health benefits like physiotherapy), but also because work was often central to an individual’s identity, provided purpose to activities, opportunities to be productive, social interactions, a distraction from health problems and even the chance for regular physical activity that helped minimize symptoms [42, 43].

These findings suggest that to help individuals sustain employment while living with arthritis it is critical to better understand the perceived balance individuals with chronic diseases have across their work, health and personal life roles, the factors associated with role balance or conflict, and the relationship of inter-role perceptions to employment outcomes. This study addresses these issues by examining three types of inter-role relationships that emerged from previous qualitative research: (a) the extent to which arthritis is perceived as negatively impacting work; (b) the extent to which work and personal life is perceived as negatively impacting arthritis symptoms and care; and (c) the extent to which work is perceived as a positive role when living with arthritis. Additionally we examine how demographic, health and work contextual factors are associated with different inter-role perceptions. Specifically, drawing on previous research we examined the degree to which arthritis was perceived as leaving too little time or energy to fulfill work demands, ways that work or personal demands conflicted with ways to effectively attend to or manage arthritis, and whether or not work was perceived as beneficial by contributing to one’s sense of identity and enjoyment or by keeping individuals active in society, potentially overcoming some of the effects of chronic arthritis symptoms. For example, greater pain and fatigue may be associated with perceptions that arthritis has a negative impact on work, whereas working unpredictable hours (e.g., shift work) or having children at home may make it difficult to manage arthritis and be associated with perceptions that work and personal demands interfere with managing one’s health. Having control over one’s work schedule or flexible work hours may mitigate the negative impact of arthritis on work and be associated with more positive perceptions of working with arthritis.

We also examine the degree to which these three inter-role perceptions (arthritis affecting work, work/personal life affecting arthritis, employment as a positive role) are associated with job stress, career satisfaction, job disruptions (e.g., arriving late, leaving early, extended breaks), absenteeism and work productivity. Poorer work outcomes are expected to be associated with greater work-health-personal life conflict whereas the perception of work as a positive role is expected to be associated with greater career satisfaction and better work outcomes.

Inter-role perceptions are examined separately for women and men. Reviews of the work-personal life balance literature find mixed evidence for gender differences [2, 4, 9]. Some studies find that women report poorer work-family fit than men, especially in families with children, while other studies find small or no significant gender effects [2, 4, 9]. Some authors have noted that, while early studies often found that women experienced more employment and family stressors than men, later research finds few differences, which may reflect changes in gender roles over time [2, 9]. In studies of individuals with arthritis, there are also mixed findings for gender and work. Some research finds no differences in employment while others find that women with arthritis are less likely to be working or more likely to need workplace accommodations [19, 40, 44]. Although men with arthritis were more likely to remain working compared to women, research has found that they reported more negative job experiences like being passed over for a promotion [19]. By examining women and men separately, we can compare similarities and differences in their perceptions of work-health-personal life balance and better understand whether arthritis is associated with unique issues related to employment.

## Participants and Methods

### Participants

Individuals 40 years of age or older with osteoarthritis (OA) or inflammatory arthritis (IA) residing in Ontario or British Columbia, Canada were recruited to study their employment experiences. Participants were recruited mainly using community advertising. Additional participants, especially those with inflammatory rheumatic diseases, which are less common, were recruited from rheumatology clinics and The Arthritis Society (TAS) website. The sample was purposive to ensure diversity across occupations and so that individuals receiving fewer health care services were not systematically excluded. To be eligible participants had to report that they had received a physician diagnosis of OA or IA (e.g., rheumatoid arthritis, psoriatic arthritis, ankylosing spondylitis), have an arthritis duration of at least 1 year, paid employment, no co-morbid conditions or injuries within the previous year causing disability (e.g., multiple sclerosis, migraine, stroke) and fluency in English.

### Procedure

Participants were screened for eligibility using a telephone-screening questionnaire. A total of 776 individuals were screened. Among them, 397 were eligible (56.1 %). Those not eligible most often had co-morbidities causing disability, injuries or recent surgery (56.8 %), were not currently employed (17.5 %), had no physician diagnosis of arthritis or were unsure of their diagnosis (13.5 %) or other considerations (e.g., English language difficulties)(12.1 %). Of the 397 participants eligible for the study, 352 were interviewed (88.7 %). Those not interviewed were repeatedly unavailable when called or not able to be contacted. Eligible participants were administered a 35–45 min, structured telephone questionnaire at a time of their choice. The telephone interview contained the primary study variables, including questions about health and arthritis severity, employment status, job type, work-health-personal life role perceptions and demographics. To reduce participant burden, provide greater flexibility for respondents in completing the study and to gather supplementary information, a shorter (20 min) self-administered questionnaire collected additional employment information on job control, career satisfaction and job stress, as well as health care utilization, which is not reported in this study. The questionnaire was mailed to respondents at the time of their telephone interview. Most self-administered questionnaires were completed and returned within 2–3 weeks of the telephone interview. In total, 352 participants completed a telephone interview and 318 participants (90.1 %) provided both telephone and self-administered data. There were no systematic differences between participants who completed or did not complete the self-administered questionnaire in terms of demographic, health or employment variables. Four interviewers conducted the telephone interviews. All completed standardized training supplemented with regular meetings and monitoring. A small honorarium (Canadian $20) was provided to participants. Ethics approval was received from the Research Ethics Board of the University Health Network, Toronto and informed written consent was obtained from all participants.

### Measures

#### Work-Health-Personal Life Balance

Participants were asked about inter-relationships among arthritis, work, and personal role demands, including negative and positive aspects of working with arthritis. Twenty-eight items were created using data from focus groups [42] and a review of arthritis-employment studies examining inter-role relationships [33, 45, 46]. Eight items asked about the impact of arthritis on work (e.g., “arthritis makes it hard to perform some of my work tasks”). Ten items asked about the impact of work and personal demands on arthritis (e.g., “working means that I have no time to look after myself properly”). Eight items asked about positive aspects of working with arthritis (e.g., “work gives me something to focus on other than my health,” “work keeps me moving and active which helps my condition”). Two items were global assessments of the inter-relationships among arthritis, work, and personal role demands (e.g., “I feel like there are not enough hours in the day to deal with work, personal demands and my health”). Items were responded to on a scale from 1 = strongly disagree to 5 = strongly agree.

#### Demographics

Data on gender, age, educational level, marital status and living arrangements (i.e., who lives with the respondent) were collected.

#### Health

Arthritis diagnosis was coded as: inflammatory arthritis (IA; e.g., rheumatoid arthritis, psoriatic arthritis), osteoarthritis (OA), or both. Disease duration was measured in number of years since diagnosis. Pain in the past month was assessed with a 0–10 visual analogue scale (VAS) (0 = no pain; 10 = worst possible pain). A homunculus displaying major joints affected by arthritis was used to calculate the total number of joints affected [47]. The Profile of Mood States (POMS) fatigue subscale asked the extent to which participants felt worn out, fatigued, exhausted, sluggish or weary in the previous month (0 = not at all; 4 = extremely) [48].

#### Employment

Participants were asked the number of hours they worked in an average week and whether their job required them to work variable or unpredictable hours (i.e., shifts) (Yes/No). Occupation was classified using the Human Resources Development Canada National Occupation Classification Matrix 2001 (Human Resources Development Canada. National occupation classification matrix 2001. URL: http://www23.hrdc-drhc.gc.ca). Occupations were classified into: 1) business, finance, administration; 2) health, teaching, sciences, arts; 3) sales, services; and 4) trades, transportation, equipment operation.

#### Work Context

Ten items asked about job control over a variety of work tasks, the pace of work, scheduling and the work environment [49]. Responses ranged from 1 = very little to 5 = very much. Internal consistency of the measure was excellent (Cronbach’s alpha = 0.90). The 12-item Workplace Activity Limitations Scale (WALS) measured arthritis-related activity limitations at work [34, 50–52]. Items assess a range of tasks (e.g., getting to, from, and around the workplace; sitting/standing for long periods; concentration at work; job scheduling). Responses were on a 4-point scale from 0 = no difficulty to 3 = unable to do. Cronbach’s alpha was 0.83. Scores were summed (range = 0–36). Participants were asked if flexible hours (“flextime”) were available to them (No = 0; Yes = 1) and whether their employer had made changes or modifications to their job to help them manage their arthritis (No = 0; Yes = 1).

#### Work Outcomes

Job stress was measured with the 15-item Chronic Illness Job Strain Scale (CIJSS) [46]. Items ask about stress related to managing symptoms at work, concerns about the future, interpersonal relationships at work, and disease unpredictability on a 5-point scale 1 = not at all stressful to 5 = extremely stressful. Cronbach’s alpha was 0.96. Career satisfaction was measured with 5-items asking about satisfaction with one’s job compared to expectations (e.g., “the progress you are making toward the goals you set for yourself in your present position”) [53]. Responses were on a 5-point scale from 1 = very dissatisfied to 5 = very satisfied. Cronbach’s alpha was 0.90. Arthritis-related job disruptions were measured with 10 items (e.g., lost work time because of arriving late/leaving early; unable to attend meetings, responsibilities, pursue a job promotion or work the shift/schedule desired; work interruptions of 20 min or more). Respondents indicated whether the job disruptions occurred related to arthritis in the previous 6 months (Y/N) and scores were summed for a total range of 0–10 [33]. Number of days absent related to arthritis in the past 6 months (including time off for appointments) was measured. Responses were collapsed into two categories: no days absent; 1 or more days absent. Participants were asked the extent to which their arthritis or its treatment resulted in being less productive at work in the past 6 months on a 5-point scale (1 = not at all; 5 = a great deal).

### Analyses

Sample descriptives, including means and standard deviations, were calculated separately for men and women. Potential gender differences in demographic, health, work context and work outcomes were tested using independent, two-tailed *t* tests. Items assessing work-health-personal life balance were examined for their distribution (floor/ceiling effects) and were correlated with one another to identify highly correlated or potentially redundant items. To explore whether the patterns of relationships among the work-health-personal life items was similar to previous qualitative research and whether items could be reduced into a smaller set of dimensions, we conducted exploratory factor analysis (EFA). Although items assessing work-health-personal life balance were generated based on concepts identified in previous research, we believed that the small number of existing studies in this area recommended caution in performing confirmatory factor analysis prematurely. Hence, we conducted EFA with single, two, three and four factor solutions. A three-factor solution was selected based on eigenvalues, factor loadings and interpretability (see results for details). Separate multivariate regression analyses for men and women were conducted to examine similarities and differences in the pattern of demographic, health and work context variables associated with inter-role perceptions. To examine the association of the three factors to employment outcomes and whether the association varied by gender, we conducted multivariate analyses that included interaction terms between gender and each of the three factors. Ordinary least squares regression examined the association of the variables to job disruptions, career satisfaction and job stress. Logistic regression examined the relationship of variables to absenteeism and ordinal logistic regression (proportional odds model) examined the association of the factors to productivity loss. Analyses examining work outcomes controlled for age, fatigue, number of joints affected by arthritis, flexible work hours, unpredictable work hours, job control, and workplace activity limitations. Analyses used SAS, version 9.0.

## Results

Table 1 presents sample descriptives for men (*n* = 104) and women (*n* = 248). There were few gender differences in demographic and health characteristics. Participants were, on average, about 51 years old, about half were married or living as married, and nearly 40 % had children living at home. There were no significant gender differences in reports of pain and fatigue or in average working hours, workplace activity limitations, perceived job control, flexible hours, or making changes to job duties. However, women were more likely to report college or university educations than men (*p* < 0.02), had a longer arthritis disease duration (*p* < 0.02) and more joints affected by arthritis (*p* < 0.01). A significantly greater proportion of women reported working in business, finance, administration or health and teaching jobs while a greater proportion of men reported working in sales and services or trades, manufacturing and transportation (*p* < 0.001). Men also were significantly more likely to report working unpredictable hours (*p* < 0.02).

**Table 1:** Sample characteristics comparing employed men (*n* = 104) and women (*n* = 248) with arthritis

	Men (*n* = 104)		Women (*n* = 248)	
	Mean (SD)	%	Mean (SD)	%
Demographics				
Age (years)	51.3 (9.6)		52.5 (8.3)	
Education				
Secondary school or some college/university		41.4		28.6*
College or university graduate		58.7		71.4
Marital status				
Married, common-law, or living as married		52.9		50.6
Single, separated, divorced, widowed		47.2		49.4
Children living in home		37.5		38.7
Health				
Diagnosis				
Osteoarthritis (OA)		47.1		52.4
Inflammatory arthritis (IA) (e.g., rheumatoid arthritis)		46.2		39.1
Both OA & IA		6.7		8.5
Arthritis duration (years)	7.1 (7.9)		9.6 (9.3)*	
Pain (range 0–10)	5.6 (2.2)		5.6 (2.2)	
Fatigue (range 0–20)	8.8 (4.2)		9.6 (4.8)	
Number of joints affected by arthritis	6.0 (3.9)		7.2 (4.8)	
Work context				
Job sector				
Business/administration		14.9		37.1***
Health/science/teaching		21.8		39.6
Sales/services		36.6		20.4
Trades/manufacturing/transportation		26.7		2.9
Average hours worked per week	37.3 (11.8)		35.4 (9.9)	
Work unpredictable hours (e.g., shift work)		50.5		35.8*
Perceived job control (range 10–50)	32.1 (10.0)		31.1 (8.8)	
Workplace activity limitations (range 0–36)	9.4 (5.1)		9.4 (5.3)	
Flexible work hours available		44.9		49.0
Job modifications made for arthritis		8.9		10.0
Arthritis-Related Work Outcomes				
Job stress (range 15–75)	40.0 (14.5)		38.9 (15.0)	
Career satisfaction (range 5–25)	16.8 (4.2)		17.6 (5.0)	
Job disruptions (range 0–10)	2.1 (2.4)		1.7 (1.9)	
Absenteeism		56.7		46.8
Productivity loss (range 1–5)	2.2 (1.0)		2.0 (1.0)	

* *p* < 0.05

*** *p* < 0.001

Many women and men reported an impact of their arthritis on work (see Table 1). There were no significant gender differences in the work outcomes. More than half of the men in the sample (56.7 %) and nearly half of the women (46.8 %) reported being absent from work in the previous 6 months because of their disease. On average, participants reported 2 arthritis-related job disruptions in the previous 6 months (e.g., arriving late/leaving early, extended breaks, missed meetings). Yet, respondents reported relatively small productivity losses because of their disease, were generally satisfied with their careers, and reported moderate amounts of job stress.

A three-factor solution with 20 items was extracted from the EFA. The three factors captured perceptions of arthritis having a negative impact on work (A → W) (8 items); work and personal life having an impact on arthritis and its management (W/P → A) (7 items); and work as a positive role (W +) (5 items). Eight items were dropped because of high correlations with other items, because they didn’t load on any of the three factors (factor loadings <0.35) or because they loaded >0.35 on more than one factor. Final factors all had eigenvalues greater than 1.0. A total mean score was calculated for items in each factor (Table 2). The factor structure and loadings were similar for men and women (data not shown) with all items having loadings of at least 0.50 on one of the three factors and no items with high factor loadings on multiple factors. Both men and women reported the highest mean scores for the factor measuring the positive benefits of working with arthritis (W +). However, an independent group *t* test found that women reported significantly greater W + agreement than men (*p* < 0.05). Cronbach’s alphas for the three factors were excellent and ranged from 0.80 to 0.89.

**Table 2:** Factor loadings, total mean scores, standard deviations and internal consistency of work-health-personal life perceptions for employed men and women with arthritis

Item	Arthritis negatively affects work (A → W) Factor loading	Work and personal life affect arthritis (W/P → A) Factor loading	Working with arthritis has positive benefits (W +) Factor loading
I don’t have as much energy at work as I would like	0.73		
My symptoms are unpredictable which creates stress	0.71		
Having arthritis means I work harder to compensate for my condition	0.75		
Arthritis makes it hard to perform work tasks	0.72		
Arthritis affects my professional image	0.70		
Arthritis makes me look less competent	0.70		
I feel guilty for not doing as good a job as I would like	0.74		
Working with arthritis means I’ve had to make tradeoffs in my life	0.59		
Working means I have no time to look after myself properly		0.67	
I feel guilty for not taking as much care of my arthritis as I would like		0.64	
Working makes it hard to attend appointments for my arthritis		0.51	
I have so much to do in my personal life I don’t have time to care for my arthritis		0.75	
I’m so tired from all the other things I have to do I don’t have energy to take care of myself		0.74	
There are not enough hours in the day to deal with work, personal demands and my health		0.74	
I worry about how I will deal with all the demands of my work, personal life and health		0.63	
Work keeps me moving and active which helps my condition			0.54
Work gives me a purpose—a reason to get up			0.78
My work is a part of who I am			0.85
Work gives me something to focus on other than my health			0.82
Work allows me to do something I really enjoy			0.80
Total mean score (SD)	3.04 (0.89)	2.89 (0.85)	3.92 (0.71)
Men	3.10 (0.86)	2.88 (0.84)	3.82 (0.71)^a^
Women	3.01 (0.91)	2.90 (0.86)	3.98 (0.70)
Cronbach’s alpha	0.88	0.87	0.81
Men	0.88	0.85	0.83
Women	0.89	0.86	0.80

Factor loadings < 0.40 are not displayed. All eigenvalues > 1.0

^a^Significant difference in W + between men and women, *p* < 0.05

Separate multivariate regression analyses for men and women examined the association of demographics, health, and work context variables to the factors in order to examine whether similar patterns or types of associations existed between men and women (see Table 3). Results indicated many gender similarities, but also some differences. Among men, greater perceptions of arthritis negatively affecting work (A → W) were associated with being older, having greater fatigue, working unpredictable hours and reporting more workplace activity limitations. Among women, greater perceived arthritis impact on work (A → W) was associated with having children living at home, greater arthritis fatigue, more workplace activity limitations and having made changes to job duties to manage arthritis.

**Table 3:** Multivariate regression analyses of demographic, health and work context variables associated with work-health-personal life perceptions for employed men and women with arthritis

	Arthritis negatively affects work (A → W)		Work and personal life affect arthritis (W/P → A)		Working with arthritis has positive benefits (W +)	
	Men β	Women β	Men β	Women β	Men β	Women β
Demographics						
Age	0.19*	−0.07	0.00	−0.08	0.13	0.00
Living with children	0.00	0.11*	0.22*	0.18**	0.11	−0.12
Health						
Pain	−0.04	0.06	0.04	0.06	0.14	−0.01
Fatigue	0.23*	0.29*	0.25	0.34***	−0.24	0.12
Number of joints affected by arthritis	0.11	0.06	−0.19	−0.02	0.35**	−0.15
Work context						
Average hours worked	0.10	−0.04	0.13	0.11	0.01	−0.10
Work unpredictable hours	0.21*	0.09	0.13	0.17**	0.09	−0.04
Job control	−0.20	0.02	−0.26*	−0.09	0.15	0.30**
Workplace activity limitations	0.34**	0.46***	0.34*	0.27**	−0.19	−0.03
Flexible work hours	−0.03	0.03	0.03	−0.02	−0.19	0.01
Job modifications made for arthritis	0.05	0.18**	−0.03	0.03	0.17	0.17*
R-squared	0.51	0.63	0.40	0.47	0.30	0.15

β = standardized regression coefficient

* *p* < 0.05; ** *p* < 0.01; *** *p* < 0.001

For men and women, greater perceived work and personal life demands affecting arthritis (W/P → A) were associated with having children at home and more workplace activity limitations. Men with lower job control also reported greater W/P → A. Women with more fatigue and who worked unpredictable hours reported greater W/P → A. Perceptions of working with arthritis as having positive benefits (W +) was associated with greater job control and having changed job duties to manage arthritis in women; and with having more joints affected by arthritis in men. Pain, hours worked per week, and having flexible hours were not associated with any of the arthritis-work-personal life perceptions for either men or women.

Regression analyses examined whether gender and the three types of work-health-personal life perceptions were associated with employment outcomes (Table 4). Analyses controlled for demographic, health and work context variables. Greater perceptions of arthritis negatively affecting work (A → W) were associated with significantly greater job stress, more job disruptions, greater absenteeism and greater perceived productivity losses. Greater perceptions of work and personal life affecting arthritis (W/P → A) were associated with more job disruptions. Greater perceptions that working with arthritis had positive benefits (W +) were significantly associated with greater career satisfaction and fewer perceived productivity losses. There were no significant main effects for gender and any of the employment variables and only one significant interaction between gender and arthritis-work-personal life perceptions. Men reporting greater work and personal life affecting their arthritis (W/P → A) had more job disruptions. For women, job disruptions did not change by perceived W/P → A.

**Table 4:** Multiple regression analyses examining the association of gender and work-health-personal life factors to job stress, career satisfaction, job disruptions, absenteeism and productivity loss

	Job Stress β	Career satisfaction β	Job disruptions β	Absenteeism OR (CI)	Productivity loss OR (CI)
Gender (female = 1)	−0.27	0.49	0.55	0.97 (0.02, 62.34)	0.07 (0.02, 2.57)
Inter-role perceptions					
Arthritis negatively affects work (A → W)	0.25*	−0.12	0.25*	2.52* (1.01, 6.24)	3.04** (1.40, 6.57)
Work and personal life affect arthritis (W/P → A)	0.04	0.07	0.31*	0.85 (0.36, 1.97)	0.94 (0.46, 1.92)
Working with arthritis has positive benefits (W +)	−0.10	0.31***	0.04	0.69 (0.34, 1.39)	0.48* (0.27, 0.86)
Interactions					
Arthritis negatively affects work (A → W)*gender	−0.16	−0.10	−0.01	0.76 (0.28, 2.06)	1.44 (0.62, 3.37)
Work and personal life affect arthritis (W/P → A)*gender	0.14	−0.07	−0.59*	1.01 (0.38, 2.70)	1.05 (0.45, 2.45)
Working with arthritis has positive benefits (W +)*gender	0.27	−0.30	−0.12	1.11 (0.47, 2.63)	1.36 (0.66, 2.78)

All analyses controlled for age, fatigue, number of joints, unpredictable work hours, job control, workplace activity limitations and flexible work hours

*OR* Odds Ratios, *CI* Confidence Interval, *β* standardized beta’s

* *p* < 0.05; ** *p* < 0.01; *** *p* < 0.001

## Discussion

Individuals living with arthritis can face challenges in working and fulfilling role demands. Previous research has focused on the negative impact of the disease on employment and not whether work and personal life roles are perceived as exacerbating arthritis symptoms or its management or whether individuals perceive positive benefits to working. Many arthritis studies also haven’t examined gender in employment experiences. This research finds similarities between women and men in their disease, work experiences and perceptions of the inter-relationships among arthritis, work and personal roles. Gender similarities and some differences in the types of factors associated with work-health-personal life balance/conflict highlight the importance of children, fatigue, unpredictable work hours, job control, and workplace activity limitations. Although data were cross-sectional, they underscore that different types of arthritis-work-personal role perceptions are variously related to a range of work outcomes. As such, the findings are an important first step in helping identify those at increased risk of work-related problems and in pointing to factors that can inform the development of interventions to improve role balance and work outcomes.

Our sample was diverse in arthritis symptoms and similar to other clinical and population health studies of arthritis and employment. Overall, women and men were similar in their health and in many aspects of work, including work hours, job control, workplace activity limitations, flexible work hours, accommodations, absenteeism, job disruptions, productivity loss, career satisfaction and job stress. However, more men in this sample reported lower education, worked unpredictable hours, and had jobs in the sales and services sector or trades, manufacturing and transportation occupations than women. Gender differences in the nature of work have been reported elsewhere. They are important to consider in future research that aims to disentangle the extent to which role perceptions are due to differences in the meaning that men and women give to their roles or whether role perceptions are largely explained by variation in role involvement like differences in job type among men and women.

Similar to other chronic diseases, arthritis studies have focused on difficulties working. This study took a broader conceptual perspective and developed a range of inter-role items. Items not only examined whether arthritis affected work, but also whether work and personal roles affected arthritis, and perceptions of the positive impact of work. Results of the factor analysis were promising for measuring arthritis-work-personal life perceptions, although additional research is needed to confirm the factor structure and assess its validity. However, the inter-role distinctions are novel and provide a more comprehensive and balanced message about working with arthritis that is important for employers, policy makers and insurers to understand. The findings also enhance conceptual models of health and employment. For example, the World Health Organization’s International Classification of Functioning, Disability and Health (WHO-ICF) recognizes the importance of participation in roles like employment but provides little theoretical guidance in this area [57]. Studies of work and family recognize the importance of perceptions of inter-role conflict but rarely consider the health of individuals [1, 5, 9, 55]. This research suggests that perceptions of arthritis-work-personal life role may be an important determinant of work outcomes in addition to demographic, health and work context factors.

Results indicated that men and women were largely similar in perceptions of the inter-relationships among arthritis, work and personal roles. They were less likely to report that work and personal roles had a detrimental impact on their health and more likely to report that working with arthritis provided positive benefits to their lives that motivated them to sustain employment. A combination of demographic, health, and work context variables were associated with arthritis-work-personal life perceptions and revealed gender similarities and differences in the pattern or types of variables related to inter-role perceptions. For both men and women, fatigue was important in understanding inter-role conflict whereas pain was not. Greater workplace activity limitations from arthritis also were associated with negative role inter-relationships for both men and women. Fatigue, which is a part of the inflammatory process, has been identified as an important problem limiting people at work [35] and found to be a predictor of difficulties working with arthritis [38, 42, 46]. However, its association with negative inter-role perceptions suggests that fatigue may make it more difficult to manage multiple role demands, leading to more negative perceptions. The association of workplace activity limitations with perceptions of arthritis negatively affecting work, as well as work negatively affecting arthritis is also of interest. Previous studies have focused on the relationship of activity limitations to job outcomes [33, 35, 50, 51]. Our findings suggest that greater attention needs to be paid to whether activity limitations at work aggravate arthritis and its management. To date, there has been little attention to fatigue and workplace activity limitations in arthritis-work interventions [54].

Having children at home was associated with perceptions that work and personal demands make it difficult to manage one’s arthritis (W/P → A) for both women and men. However, women also reported that their arthritis had a negative impact on work (A → W) when children were living at home. Although it’s not clear why, it may be that women in the study had more responsibilities for children living at home than their male counterparts. As a result, the presence of children had a wider impact on role inter-relationships.

Among women, there was a significant relationship between job modifications and perceptions that arthritis affected work (A → W) and that work was beneficial when living with arthritis (W +). Given the cross-sectional nature of the study, the direction of the findings cannot be established and longitudinal research is needed. Women and men reported making a similar number of job modifications. However, having to make changes in order to stay working may have been appraised as a signal of the negative impact of their disease among women, as well as indicative of the supportiveness of their workplace. Future research needs to examine in more detail the type of modifications made by women and men in response to job difficulties.

Job control was associated with arthritis-work-personal life perceptions for both men and women. For men however, lower job control was significantly related to greater perceptions that work and personal life negatively affected arthritis (W/P → A). For women, perceptions of work as a positive role (W +) were associated with greater job control. Theories and research have underscored the importance of job control to employment outcomes [55]. The current study suggests that its role may vary for men and women with a lack of control being particularly relevant for men in understanding conflict in balancing work and health roles.

Similar to low job control, working unpredictable hours was associated with greater perceptions of arthritis negatively impacting work among men (A → W). Among women the perception was not that unpredictable hours created problems in the workplace. Instead, it was associated with difficulties managing one’s disease (W/P → A). The findings suggest that additional research is needed to examine whether similar experiences at work may be appraised differently by women and men in terms of the direction of their role perceptions.

Of interest was that, among men, having more joints affected by arthritis was associated with positive perceptions of working. This finding is counterintuitive given that one might expect that greater joint involvement would increase the risk of problems working and could potentially aggravate arthritis. Additional research is needed to confirm and further explore this finding. However as noted earlier, men in this study reported jobs that were potentially more physically active than women. Opportunities to be active at work was reported as a perceived benefit of working with arthritis (W +) not only in this study, but also in qualitative research [42, 43]. Previous research also has linked physical activity to better health and well-being in arthritis [56]. It may be that some men with more arthritis joint involvement reaped benefits from physically active jobs or that too little or too much physical activity makes working with arthritis difficult, while moderate physical demands are perceived as having benefits for arthritis.

Finally, this research examined the relationship of gender and role perceptions to arthritis-related absenteeism, job disruptions, productivity loss, career satisfaction and job stress. Because the study was cross-sectional, it is difficult to determine whether the work outcomes that were examined contributed to differing role perceptions or whether some types of role perceptions may subsequently impact work outcomes. For example, positive perceptions of working with arthritis (W +) were associated with lower perceived productivity loss and greater career satisfaction. While it may be that individuals who had little impact of their disease at work were able to see greater benefits to employment, it also may be that having a positive attitude toward working with arthritis resulted in changes to behaviours and attitudes that subsequently impacted productivity and career satisfaction. Longitudinal research is needed to disentangle the direction of findings and to better understand the conceptual linkages of arthritis-work-personal life perceptions and health and work context factors, including whether inter-role perceptions modify the role of symptoms and work context factors in predicting employment outcomes.

Also important to note was the relative absence of gender differences. We found only one significant interaction effect indicating that, among men, greater perceptions of work and personal life affecting arthritis (W/P → A) was related to more job disruptions. These findings are in keeping with recent studies finding few gender differences in work-family perceptions [2, 9]. They may also indicate that, although there are sex differences in prevalence of arthritis with more women being diagnosed with many types of rheumatic conditions, there are few gender differences in how women and men perceive the inter-relationships among their arthritis, work and personal roles and the impact of these roles on employment.

Several limitations to this research need consideration. The study was cross-sectional and the direction of findings cannot be fully determined. Additional research is needed to examine whether role perceptions reflect the difficulties individuals with arthritis have in working or whether they may also act as risk factors, signalling potential problems with employment in the future. This research also utilized a convenience sample of employed individuals. Examining additional outcomes like giving up employment and comparing individuals who are working with those not in the labour force in additional samples would provide further insights into the determinants and consequences of role conflict. As noted earlier, research is also needed to confirm the factor structure of the three role dimensions. Finally, men and women reported working in different job sectors, which may explain the findings. Attention to a wider range of work context factors may illuminate in greater detail gender similarities and differences in arthritis-work-personal life perceptions.

In conclusion, this study highlights a range of perceptions about working with a chronic illness, including positive perceptions of the role of employment in people’s lives. Although many individuals with arthritis reported some negative impact of their disease on work, they believed that working with arthritis was positive in a variety of ways. Moreover, men and women were often similar in their appraisals of the inter-relationships among arthritis, work, and personal life roles. Some gender differences in the factors associated with inter-role perceptions and the association of arthritis-work-personal life perceptions with job outcomes highlights the need for continued examination of health and work context factors that may enhance work outcomes or act as barriers to employment.
